# Medical Matters in Havana

**Published:** 1887-04

**Authors:** Frank S. Johnson

**Affiliations:** Havana, Cuba


HAVANA, CUBA, February 20th, 1887.

*To the Editor of the* CHICAGO MEDICAL JOURNAL AND EXAMINER:

Of the many who seek warm winter climates, a small proportion go to the island of Cuba. Havana is usually the objective point. Cuba lies under the tropic of Cancer, its southern portion being within the tropics. Along its northern coast flows that great ocean river, the Gulf Stream. Its width opposite Havana is 90 miles; the temperature of its water about 78° F.

The Cuban winters are, therefore, always warm. They are also dry. During our brief stay on the island, the thermometer has registered 80° F., or higher, in the shade, at mid-day. During the day the air is comfortable; at night it is damp; the dews are very heavy, and the air seems chilly. The prevailing winds during the winter months are from the northeast. The city of Havana, situated upon its deep bay, with its narrow entrance, is built upon soft, porous, coral rock. Its site is nearly level. The older part of the city is upon the water's edge. It was once surrounded by a wall and fortifications. The houses in this portion are small, and the rooms are badly ventilated, some, even, being without windows; and they are often much overcrowded. The classes inhabiting this district are negroes and the poorest whites. The streets, with few exceptions, are unpaved. The newer and better-built portions are back from the bay, upon slightly higher ground. In these the streets are well paved with stone. The houses are larger; the rooms have high ceilings, and are well ventilated. But even here there are many unsanitary conditions.

The house plan in Cuba is an inheritance. It came from Old Spain three hundred years ago. The rooms open upon a court; there is but one entrance to the house; the drainage often reaches the street by an open gutter. Privy vaults are still used, and are within the dwelling. The hall serves as carriage-room and stable.

The water supply of Havana is scanty. Water is brought to the city partly in canals and open aqueducts, and partly in pipes. In many parts of the city it does not rise above the first floor. It is obtained from small streams, and its quality is not uniform.

The city is badly drained. Large areas are unsewered, especially in the older and unpaved districts. In the portions that are supplied with sewers, the drainage is imperfect. During the winter the sewage stagnates in the conduits, because of lack of water to carry it on. Where the drainage is upon the surface, the fluids dry up or are absorbed by the underlying porous rock. In the wet season the heavy rains flood the sewers, and the accumulated sewage is washed into the bay, where there is no constant current, and a tide of only 18 or 20 inches. Dilution is effected very slowly, and the contaminated waters near the stream are washed up and down along the line of the wharves. The water at a distance from the shore, out in the bay, is comparatively clean.

The navy yard is contiguous to a long line of wharves, and adjoining it is the Military Hospital, with capacity of 2,000 beds. The hospital is never entirely free from yellow fever, and through its sewage adds to the infectious material brought to the bay by the city conduits. The air along the shore is more or less polluted. During the day the odors are less disagreeable, and there is little or no danger then; but it is not prudent for strangers to be on the wharves, even in winter, after the sun has set and the heavy dews have begun to fall.

Vessels that discharge and take on cargoes at the wharves do so at the risk of becoming infected. They often lie one, two, and sometimes three weeks with their hatches open and their holds exposed nightly to the damp, foul air. To those anchored in the clean water, off from the shore, the risk is very slight; while to vessels that arrive and leave upon the same day, as is done by the United States mail steamers running between Tampa and Havana, the risk is reduced to the minimum; they are comparatively safe, even during epidemics. A brief survey shows that the sanitary conditions of Havana are not good. Its death rate is 43 per 1,000. The disease most dreaded by strangers is yellow fever. Yet the yearly mortality from yellow fever is less than from phthisis. Yellow fever is endemic and nearly always present, but the number of cases in winter is very small. They originate almost without exception in the older and most uncleanly portions of the city, in houses almost incapable of ventilation, in rooms that have probably been infected by repeated epidemics; some cases also occur in the shipping. For the stranger in Havana the conditions are different. The best hotels are located in the most healthful parts of the city, and are furnished with modern improvements. Reasonable attention to diet and precaution against exposure to the damp night air and the hot noon-day sun render the winters perfectly safe.

The medical institutions of Havana are of interest, and I am indebted to the kindness of W. D. M. Burgess for the opportunity of seeing the hospitals of this tropical city.

Yours truly, FRANK S. JOHNSON, M. D.

